# Influence of donor–recipient sex mismatch on long-term survival of pancreatic grafts

**DOI:** 10.1038/srep29298

**Published:** 2016-07-12

**Authors:** Zhiwei Li, Shengmin Mei, Jie Xiang, Jie Zhou, Qijun Zhang, Sheng Yan, Lin Zhou, Zhenhua Hu, Shusen Zheng

**Affiliations:** 1 Division of Hepatobiliary and Pancreatic Surgery, Department of Surgery, First Affiliated Hospital, School of Medicine, Zhejiang University, Key Laboratory of Combined Multi-organ Transplantation, Ministry of Public Health, Key Laboratory of Organ Transplantation, Zhejiang Province, Hangzhou, China; 2 Collaborative Innovation Center for Diagnosis and Treatment of Infectious Diseases, Hangzhou, China

## Abstract

To assess the role of sex mismatch on graft survival after pancreas transplantation. We evaluated 24,195 pancreas-transplant recipients reported in the Scientific Registry of Transplant Recipients over a 25-year period. Pancreatic graft survival (PGS) was analyzed according to donor–recipient sex pairing using Kaplan–Meier estimations. Hazard ratios were estimated using Cox proportional hazard models. A total of 14,187 male and 10,008 female recipients were included in final analyses. Mean follow-up was 8.3 ± 5.7 years. In multivariate analyses, neither recipient sex nor donor sex was associated with pancreatic graft failure (PGF), but donor–recipient sex mismatch (regardless of recipient sex) was an independent predictor of PGS (HR, 1.09; 95% CI, 1.04–1.14; p < 0.001). Compared with M → M sex-matched recipients in univariate analyses, M → F and F → M sex mismatches were associated with an increased risk of PGF. Adjustment for significant recipient and donor factors eliminated the association between F → M sex mismatch and PGF (HR, 1.02; 95% CI, 0.93–1.10; p = 0.752), but not M → F (1.09; 1.02–1.17; 0.020). Stratified analyses suggested that the negative effect of donor–recipient sex mismatch could be neutralized in older patients. These findings suggest that donor–recipient sex pairing should be taken into consideration in organ-allocation strategies.

Sex plays an important part in the outcomes of individuals receiving solid-organ transplants^1^,^2^,^3^,^4^,^5^. In liver-transplant recipients, female recipients and donors have been found to be associated with an increased prevalence of death and graft failure^4^. More recent studies have highlighted the importance of donor–recipient sex pairing in organ transplantation.

Several reports on liver transplantation have shown that recipients with sex-mismatched donors have an increased risk of graft failure compared with those with sex-matched donors^6^,^7^,^8^,^9^. In heart-transplant recipients, donor–recipient sex mismatch also increases the risk of death (mainly during the first month) and in patients with pulmonary gradient >13 mmHg^10^. Kaufman and colleagues found that female donors were associated with graft loss after pancreas transplantation, and that donor sex was integrated into the formula for the Pancreas Donor Risk Index (PDRI)^11^. A study from Norway confirmed recipient sex not to be associated with pancreatic graft survival (PGS) or patient survival^12^.

However, all of these reports looked only at the impact of donor sex or recipient sex. Also, the results of studies analyzing the influence of associations between donor sex and recipient sex on PGS in small-sample cohorts or in single-center studies have been contradictory^13^,^14^,^15^,^16^,^17^.

We hypothesized that the types of donor–recipient sex matching are relevant in predicting outcomes of pancreatic grafts. Accordingly, using a large national registry database, we sought to clarify and quantify the influence of donor–recipient sex pairing on the outcomes of pancreas transplantation.

## Methods

### Data sources

This study was based on the Scientific Registry of Transplant Recipients (SRTR), which comprises data on all donors, waiting-list candidates, and transplant recipients in the USA, and which is submitted by members of the Organ Procurement and Transplantation Network (OPTN). The Health Resources and Services Administration and the US Department of Health and Human Services oversee the activities of the OPTN and SRTR contractors^18^. The study protocol was approved by the Ethics Committee of Zhejiang University (Hangzhou, China).

### Study cohort

**Figure 1:**
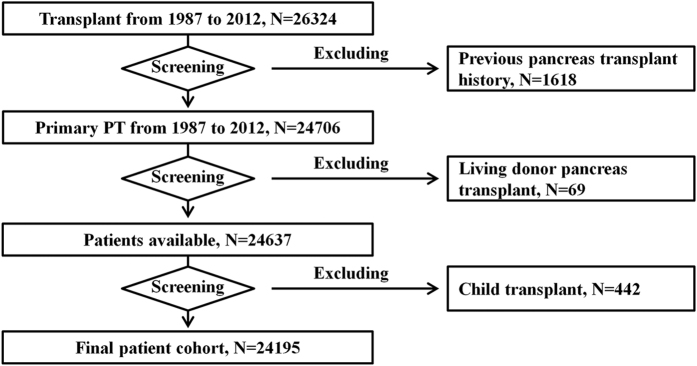
Patient selection. PT, pancreas transplantation.

Individuals who received a pancreas transplant from 1 October 1987 to 30 September 2012 formed the study cohort. Patients with a history of pancreas transplant, aged <18 years, or who had received a graft from a live donor were excluded from analyses. The subject-selection process is depicted in Fig. 1.

The following data of recipients were extracted: age; ethnicity; transplant type; year of transplant; height; weight; Body Mass Index (BMI); human leukocyte antigen (HLA) mismatch; panel-reactive antibody (PRA); type of exocrine drainage; type of endocrine drainage; time since onset of diabetes mellitus (DM); date of transplant; date of graft failure; date of death; date of final follow-up. Ethnicity was grouped as “Caucasian”, “African–American”, “Asian/Pacific Islander”, “Hispanic” and “other”. Types of transplant were categorized as “simultaneous pancreas–kidney transplantation” (SPK), “pancreas after kidney transplantation” (PAK), and “pancreas transplantation alone” (PTA). BMI classes were as defined by the World Health Organization (in kg/m^2^): underweight (<18.5), normal (18.5–24.9), and overweight (≥25.0).

### Donor variables

“Sex mismatch” was defined as a female donor to a male recipient (F → M) or a male donor to a female recipient (M → F). “Sex match” was defined as a female donor to a female recipient (F → F) or a male donor to a male recipient (M → M). The PDRI was calculated using the formula established by Axelrod and colleagues^11^. Missing values for pancreas preservation time were imputed with median times. Other relevant donor data were age, ethnicity, height, weight, BMI, creatinine concentration in serum, cause of death, history of hypertension, and donation after cardiac death. Cause of death was grouped as “anoxia”, “cerebrovascular accident”, “head trauma” and “other”. Cutoff values for the variables mentioned above that seemed to be implausible were: recipient/donor BMI <10 kg/m^2^ or >40 kg/m^2^; recipient/donor height <100 cm or >240 cm; recipient/donor weight <20 kg or >180 kg. Observations involving these implausible values were classified as “missing”. Sensitivity analyses when comparing multivariate models used a case-wise deletion method for missing values so that the imputation did not change interpretation of the final results.

### Analyses of outcomes

Primary outcome was the survival time of the pancreatic graft. The main predictor of our study was donor–recipient sex mismatch. Analyses focused on the association between donor–recipient sex mismatch and outcome of pancreatic grafts. Follow-up data were collected by electronic means every 3–6 months during the first year and then yearly. Endpoint of analyses of graft survival was pancreatic graft failure (PGF), the date of which was defined in the SRTR as the date of pancreas re-transplant, transplant pancreatectomy, or return to exogenous insulin.

### Statistical analyses

Results are expressed as the mean ± standard deviation for continuous variables, and counts and percentages for categorical variables, with one-way analysis of variance F-test and chi-square test being used, respectively, to test whether these characteristics differed. Then, time-to-event analyses were undertaken, and patients were censored at the time of their final follow-up. Kaplan–Meier estimates were assessed for PGF, and the log-rank test was used for comparison. Significance was assessed at 0.05 (two-sided). Cox proportional hazards models were used to estimate hazard ratios (HRs) and undertake tests. A multivariable Cox model was used to assess the factors associated with PGF. Multivariable models were derived using backward stepwise selection of variables with a cutoff for inclusion of p = 0.1. HRs and 95% confidence intervals (CI) were assessed for each variable included in the multivariate model. Potential time-dependent effects were evaluated and test statistics were based on Schoenfeld residuals^19^. Time-dependent effects were modeled by extending the Cox model with introduction of cubic spline functions^20^. All analyses were done by SAS v9.2 (SAS Institute, Cary, NC, USA).

## Results

### Population characteristics

A total of 24,195 adult recipients undergoing primary pancreas transplantation were involved in the final analyses. Mean age of the cohort was 40.0 ± 8.5 years, and the male-to-female ratio was 59%:41%. Demographics and clinical characteristics of patients are shown in Table 1. Mean follow-up was 8.3 ± 5.7 years.

There was no significant difference in ethnicity between male and female recipients (p > 0.05). However, female recipients were younger and had lower height, weight, and BMI than male recipients. A greater proportion of males underwent SPK, and had HLA mismatch >2/6. More women had a PRA > 20% at the time of pancreas transplantation. DM duration in men was slightly longer than that in women. Surgical procedures, transplantation date after 1998, and the PDRI were not significantly different between the two groups (p = 0.830).

There were 16,270 (67%) male donors and 7,923 (33%) female donors (Supplemental Table 1). Among these donors, female donors were significantly older than male donors (p < 0.001), and a greater proportion of female donors had a history of hypertension. Though female donors were shorter and weighed less than male donors, the difference in BMI between the two groups was not significantly different (p = 0.870). Compared with male donors, female donors were more likely to die of cerebrovascular accident or stroke, had a lower level of creatinine in serum, and slightly longer pancreas preservation time. There were no significant differences in the proportion of donors who suffered cardiac disease-based death (p = 0.159).

### Impact of differences in the sex of donors and recipients on PGS

Estimated PGS using Kaplan–Meier survival curves was significantly better in male recipients (log rank, p < 0.001) (Supplemental Figure 1A) or in recipients with male donor grafts (log rank p = 0.032) (Supplemental Figure 1B). Univariate analyses revealed male recipients (HR, 0.90; 95% CI, 0.86–0.94; p < 0.001) and male donors (0.95; 0.90–0.99; 0.033) to have a decreased risk of PGF when compared with female recipients and female donors, respectively. However, after adjustment for other recipient- and donor-related factors, the association between recipient sex or donor sex and PGF disappeared (Supplementary Table 2).

### Impact of donor–recipient sex mismatch on PGS

**Figure 2:**
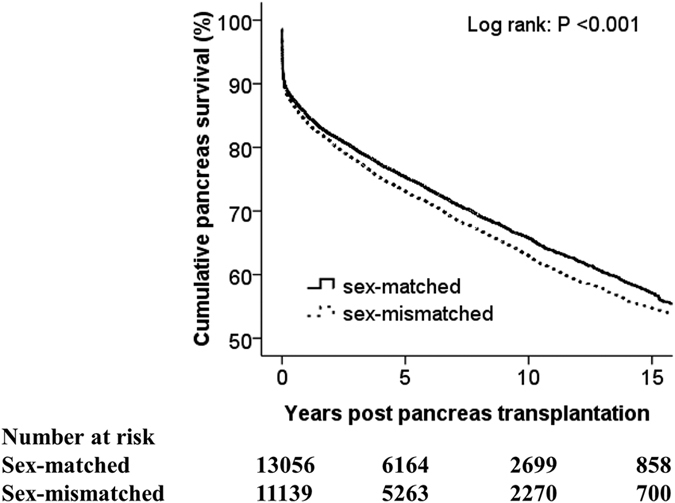
Kaplan–Meier pancreatic-graft survival curves for recipients with and without sex-mismatched donors.

Female recipients were more than twice as likely as male recipients to receive a sex-mismatched graft (66.1% *vs*. 31.9%, p < 0.001). Kaplan–Meier graft-survival curves for the donor–recipient sex-matched group and donor–recipient sex-mismatched group are shown in Fig. 2. Prevalence of PGS in the donor–recipient sex-matched group (regardless of recipient sex) was 85%, 75%, 65%, and 57% at 1, 5, 10, and 15 years, respectively, compared with 84%, 73%, 63%, and 55% in the donor–recipient sex-mismatched group at identical time points (log rank p < 0.001). Compared with recipients with a sex-matched graft, recipients with a sex-mismatched graft had a 9% higher risk of PGF by multivariate analyses (HR, 1.09; 95% CI, 1.04–1.14; p < 0.001). Further research on the cause of PGF showed that, in the 11,139 recipients with a sex-mismatched graft, 1,133 (10.2%) recipients developed acute rejection of pancreatic allografts, but the difference with those with a sex-matched graft was not significant (10.3%) (p = 0.750).

**Figure 3:**
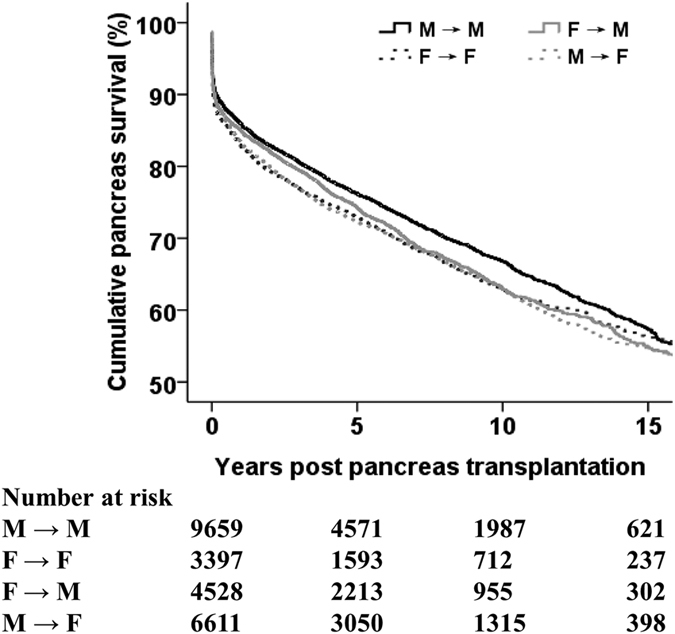
Kaplan–Meier pancreatic-graft survival curves by donor–recipient sex pairing.

Recipients were divided into four groups: M → M, F → F, M → F, and F → M. Kaplan–Meier estimates of PGS according to donor–recipient sex pairing are shown in Fig. 3. Prevalence of PGS at 1, 5, 10, and 15 years was 86%, 76%, 67%, 57% for the M → M matched group; 83%, 73%, 63%, and 56% for the F → F matched group; 85%, 74%, 63%, and 55% for the F → M mismatched group; and 83%, 72%, 63%, and 54% for the M → F mismatched group (p < 0.001). Then, all HRs involving donor–recipient sex mismatching in subsequent analyses were reported using M → M as the reference group. In univariate analyses, M → F mismatched recipients (HR, 1.15; 95% CI, 1.08–1.21; p < 0.001) and F → M mismatched recipients (1.10; 1.03–1.17; 0.004) were at an increased risk of PGF compared with M → M matched recipients (Table 2). After adjustment for recipient- and donor-related factors, there was no association between F → M mismatch and PGF, but M → F mismatch remained predictive of PGF (HR, 1.09; 95% CI, 1.02–1.17; p = 0.020) (Table 2). Other independent predictors for PGF were recipient age (per year: HR, 0.97; 95% CI, 0.97–0.98; p < 0.001), recipient BMI (1.02; 1.01–1.03; <0.001), transplant type (PAK/PTA *vs*. SPK: 1.30; 1.26–1.34; <0.001), and the PDRI (1.51; 1.43–1.60; <0.001) (Table 2).

### Stratified analyses of the impact of sex mismatch on PGS

**Figure 4:**
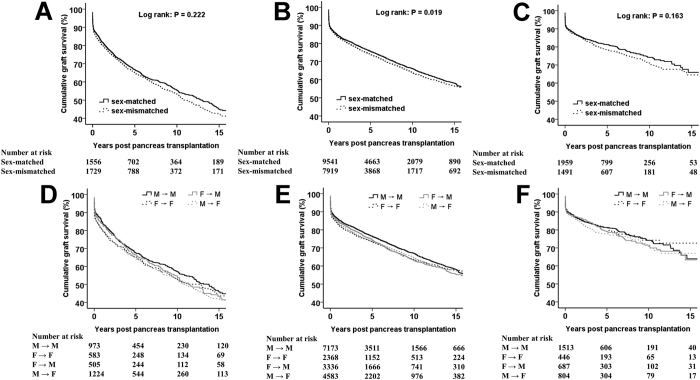
Estimated survival of pancreatic grafts stratified by donor–recipient sex pairing in transplant recipients aged 18–30 (**A,D**), 31–50 (**B,E**), and ≥51 years (**C,F**).

In subgroup analyses to evaluate further the association between donor–recipient sex pairing and PGF, an important effect modification by recipient age and recipient BMI was observed. Figure 4 shows Kaplan–Meier survival curves for each donor–recipient sex pairing in the age groups of 18–30 years, 31–50 years, and ≥51 years, respectively. Univariate analyses revealed that recipients in both donor–recipient sex-mismatched groups experienced an increased HR of PGF compared with M → M matched recipients, but this effect was not seen among recipients aged >50 years (Table 3). After adjustment for other factors related to PGF only M → F mismatched recipients in 18–30 and 31–50 age groups continued to experience an increased HR of PGF (Table 3). Among recipients aged >50 years, an increased risk of PGF in donor–recipient sex-mismatched groups (regardless of recipient sex) was not observed (Table 3). In stratified analyses according to recipient BMI, we obtained similar results. That is, only M → F sex-mismatched recipients in the normal group (18.5–24.9 kg/m^2^) experienced an increased HR of PGF, and this phenomenon was not seen among underweight (<18.5 kg/m^2^) or overweight (≥25.0 kg/m^2^) recipients (Supplemental Figure 2 and Supplemental Table 3).

The time-dependent HR of PGF for donor–recipient sex mismatch compared with sex matching by Cox-derived estimates was almost constant over time after pancreas transplantation. Using an illustration of the impact of donor–recipient sex-mismatched allocation, 10-year PGS was estimated from the final Cox model in different clinical scenarios (Supplemental Table 4). For any given combination of independent risk factors of PGF, older recipients (age > 50 years) with donor–recipient sex-mismatched donor grafts were associated with gain of ≈10% in 10-year PGS.

## Discussion

The present study is the first to show that neither donor sex nor recipient sex is associated with PGF in multivariate analyses, but that donor–recipient sex mismatch is associated with PGF in all types of pancreas transplantation. The large size of the adult pancreas transplantation population always allow for detection of a small but statistically significant difference in graft survival and patient mortality. Recipients with sex-mismatched donors had a higher risk of PGF of 9% when compared with those with sex-matched donors. This difference remained highly significant after adjustment for covariates by Cox regression analyses. Further stratified analyses demonstrated that M → F mismatch is an independent predictor of PGF, and that other risk factors are: young recipients; a high PDRI, PAK or PTA; overweight recipients. Notably, a less favorable outcome for recipients of a pancreas transplant undertaken with donor–recipient sex-mismatched donor grafts was not observed in older recipients (age, ≥51 years), who achieved excellent results irrespective of donor–recipient sex pairing.

PGS continues to improve with better procurement of grafts and immunosuppression regimens^14^,^21^,^22^. More risk factors associated with PGF have been recognized: type of pancreas transplantation; type of exocrine drainage; recipient age; donor age^23^,^24^. However, very little research has focused on the relationship between sex differences and patient survival. Colling and colleagues reported that recipient sex affected outcome after pancreas transplantation, and that a higher prevalence of early (<6 months) PGF was observed in women^15^. Schaffer and colleagues found an increased risk of organ rejection in female donors after pancreas transplantation^17^. To optimize organ utilization due to the severe shortage of donors worldwide, donor-related factors have been studied widely for their impact on graft function. Accordingly, Axelrod and colleagues introduced a formula based on donor age, sex, height, ethnicity, BMI, cause of death, pancreas preservation time, donation after cardiac death, and creatinine level in serum as a quantitative measure of graft quality which is known as the PDRI^11^. Their data suggested that female donors have a negative impact on PGS.

Using the SRTR, we found that male recipients or recipients with male donors shared significantly better Kaplan–Meier-estimated PGS, which was consistent with Axelrod’s results to a certain extent, but that neither of them was an independent predictor of PGS after adjustment of other donor- and recipient-relevant factors. Two reasons for this difference between our study and previous works can be postulated: (i) most previous analyses were single-center studies, and did not account for the multiple characteristics of donors or the large research population. Meanwhile due to limited sample cohorts in the single-center studies, they likely yielded contradictory results or could not find subtle differences; (ii) our multivariate analyses involved not only donor factors, but also transplant-related and recipient factors, such as transplant type and year of transplant, which was confirmed to be associate with pancreatic graft outcome by Bedat and his colleagues^5^.

Further research about the impact of donor–recipient sex mismatch PGS was conducted. Several studies have focused on the influence of donor–recipient sex mismatch on the outcomes of liver, kidney and heart transplantation^10^,^25^,^26^,^27^,^28^,^29^,^30^,^31^, but few authors have looked at on this aspect in pancreas transplantation. Large-scale analyses of data from the United Network for Organ Sharing have revealed that donor–recipient sex mismatch is an independent predictor of graft loss among liver-transplant recipients^25^. Grat and colleagues also found that M → F sex mismatch tends to reduce graft survival in liver-transplant recipients infected with the hepatitis-C virus^26^. Gratwohl and his colleagues reported that sex mismatched recipients was associated with an increased risk of renal graft failure^30^, while Kim and his colleagues indicated that sex mismatched recipients had an increased short-term risk but no long-term risk of graft failure when compared with sex matched recipients^29^. A study by the International Society of Heart Lung Transplantation suggested that heart-transplant recipients receiving organs from same-sex donors had significantly improved graft survival^32^. Kaczmarek and colleagues also found that male recipients with female allografts had the worst prevalence of survival, and that the prevalence of survival for remaining pairings was similar in heart transplantation^27^. We found that donor–recipient sex mismatch was associated with PGF, and that M → F sex mismatch is an independent risk factor for PGF (M → M matched group as a reference). However, there was no differences in PGF among M → F sex mismatched recipients, F → M mismatched recipients and F → F matched recipients. Further stratified analyses eliminated the association between M → F sex mismatch and PGF among underweight (BMI < 18.5 kg/m^2^), overweight (BMI ≥ 25.0 kg/m^2^), and older recipients (age > 50 years). This is the first study on PGS after pancreas transplantation looking specifically at sex mismatch with respect to donor and recipient.

Mechanisms by which donor–recipient sex mismatch could affect graft survival after transplantation could include hormonal differences, genetic differences, and immunologic factors. Ruhe and colleagues reported that the extent of impact of age on pancreatic endocrine function (e.g., insulin secretion, glucose sensitivity of the islets of Langerhan) was distinctly different^33^. In addition, donor–recipient sex mismatch has been linked with immune rejection due to the male H-Y minor histocompatibility antigen, especially for M → F mismatch^34^. Tan and colleagues found M → F sex mismatch triggered *de novo* production of H-Y alloantibodies that led to acute rejection of grafts in renal transplantation^35^. This hypothesis is consistent with our results: M → F sex-mismatched recipients (adjusted HR, 1.09) had the worst PGS. Through further analyses stratified by significant predictors of PGS, recipient age, and BMI, we found that the negative influence of M → F sex mismatch on PGS deteriorated among young recipients (adjusted HR, 1.27) and normal-weight recipients (adjusted HR, 1.12). However, there was no significant difference in episodes of acute rejection after pancreas transplantation between sex-mismatched recipients and sex-matched recipients.

Taken together, these data suggest that donor–recipient sex mismatch represents a surrogate marker of inferior PGS. Notably, the estimated higher HR of PGF for donor–recipient sex mismatch *versus* donor–recipient sex match was almost constant over time after pancreas transplantation. That is, donor–recipient sex mismatch may injure the health of recipients during their entire post-transplant lives. The better outcomes observed in older recipients with sex-mismatched donors suggest that the negative effect of donor–recipient sex mismatch could be neutralized by optimal selection of recipients and appropriate care before transplantation.

Currently, donor–recipient sex pairing is not taken into consideration during donor–recipient matching in pancreas transplantation. Findings of the present study suggest that donor–recipient sex matching could be integrated into the allocation criteria of donor organs for pancreas transplantation. The worldwide shortage in the supply of donor organs and tissues is becoming more pronounced, so the program of optimal allocation of organs is based on a lower prevalence of waiting-list death and longer survival of recipients. Most donors are male and donor–recipient matching must account for blood type, HLA, and PRA, and accepting or declining a pancreatic allograft is a difficult decision for any candidate. Therefore, further studies are required to determine the utility of such a strategy.

The registry nature of the study by using the SRTR database is related to several limitations. We cannot evaluate the severity of pretransplant disease and detail posttransplant immunologic regimen. Such influence cannot be excluded from these data because selection of immunosuppressive agent is not based on gender in common clinical practice. Then, surgery related factors that could not be evaluated in detail may have a sex-specific impact on perioperative survival after pancreas transplant just as in other surgery in adults^36^. In addition, the SRTR data are based on information collected for all pancreas transplants performed in the United States and individual reporting bias by transplant centers may lead to under-estimation or over-estimation of results; however, we believe that this bias would be similar between the groups that were compared. We were unable to accurately evaluate other outcome variables, such as technical failure, immune mediated graft loss, and rejection rates, which could be used to assess the function of pancreatic graft more comprehensively, because those data are notoriously poorly reported in SRTR data. However, in an effort to provide better understanding of the sex effect on PGS, we studied both occurrence of acute rejection and perioperative mortality, thus providing a good reading of the effect of sex match or sex mismatch on PGS.

In conclusion, donor–recipient sex mismatch is associated with PGF after pancreas transplantation. In particular, M → F sex mismatch should be evaluated with caution because it may be associated with the worst PGS. However, this negative effect of donor–recipient sex mismatch could be eliminated in older recipients. Overall, these findings suggest that to allocate scarce pancreatic grafts to appropriate candidates, donor–recipient sex pairing should be considered in the allocation strategies for pancreatic allografts.

## Additional Information

**How to cite this article**: Li, Z. *et al*. Influence of donor–recipient sex mismatch on long-term survival of pancreatic grafts. *Sci. Rep.* **6**, 29298; doi: 10.1038/srep29298 (2016).

## Supplementary Material

Supplementary Information

## Figures and Tables

**Table 1:** Characteristics of pancreas-transplant recipients from 1 October 1987 to 30 September 2012.

	Total(n = 24195)	Male recipients(n = 14187)	Female recipients(n = 10008)	*P*
Age in years at transplant: mean (SD)	40.0 ± 8.5	40.7 ± 8.3	38.9 ± 8.7	<0.001
Ethnicity: N (%)				
Caucasian	19,641 (81.2)	11,458 (80.8)	8183 (81.8)	0.051
African–American	2667 (11.1)	1591 (11.2)	1076 (10.8)	0.261
Asian/Pacific Islander	222 (0.9)	112 (0.8)	110 (1.1)	0.101
Hispanic	1534 (6.3)	952 (6.7)	582 (5.8)	0.055
Other	131 (0.5)	74 (0.5)	57 (0.6)	0.658
Type of transplant: N (%)				
SPK	18,134 (74.9)	10,979 (77.4)	7155 (71.5)	<0.001
PAK	2115 (8.8)	1233 (8.7)	882 (8.8)	0.747
PTA	3096 (12.8)	1492 (10.5)	1604 (16.0)	<0.001
Unknown	850 (3.5)	483 (3.4)	367 (3.7)	0.287
Transplant before 1998: N (%)	6773 (28.0)	3915 (27.6)	2858 (28.6)	0.103
BMI: mean (SD)	24.5 ± 4.6	24.8 ± 4.3	24.3 ± 4.9	<0.001
Height in centimeters: mean (SD)	170 ± 10	175 ± 8	163 ± 8	<0.001
Weight in kilograms: mean (SD)	71.4 ± 14.7	76.5 ± 14.2	64.2 ± 12.2	<0.001
HLA mismatch > 2/6: N (%)	22,049 (91.1)	13,052 (92.0)	8997 (89.9)	< 0.001
PRA% > 20%: N (%)	3196 (13.2)	1326 (9.3)	1870 (18.7)	< 0.001
Exocrine drainage: N (%)				
Bladder drainage	8506 (35.2)	4925 (34.7)	3581 (35.8)	0.087
Enteric drainage	14,768 (61.0)	8726 (61.5)	6042 (60.4)	0.075
Others	921 (3.8)	536 (3.8)	385 (3.8)	0.786
Endocrine drainage: N (%)				
Systemic system	20,194 (83.5)	11,871 (83.7)	8323 (83.2)	0.292
Portal system	3669 (15.1)	2140 (15.1)	1529 (15.3)	0.689
Other	332 (1.4)	176 (1.2)	156 (1.6)	0.037
Years since DM onset: mean (SD)	26.2 ± 8.5	26.4 ± 8.3	26.1 ± 8.7	0.039
Follow-up in years: mean (SD)	8.3 ± 5.7	8.3 ± 5.6	8.3 ± 5.8	0.229
PDRI: mean (SD)	1.16 ± 0.43	1.16 ± 0.43	1.16 ± 0.43	0.830

SD, Standard deviation; SPK, simultaneous pancreas–kidney transplantation; PAK, pancreas after kidney transplantation; PTA, pancreas transplantation alone; BMI, Body Mass Index; HLA, human leukocyte antigen; PRA, panel-reactive antibody; DM, diabetes mellitus; PDRI, Pancreas Donor Risk Index.

**Table 2:** Evaluation of pancreatic-graft survival using univariate and multivariate Cox regression.

Covariates	Univariate HR(95% CI)	*P*	Multivariate^1^ HR(95% CI)	*P*
M → M match (reference)	1.00		1.00	
F → F match	1.14 (1.06–1.22)	<0.001	0.95 (0.87–1.05)	0.295
F → M mismatch	1.10 (1.03–1.17)	0.004	1.02 (0.93–1.10)	0.752
M → F mismatch	1.15 (1.08–1.21)	<0.001	1.09 (1.02–1.17)	0.020
PDRI			1.51 (1.43–1.60)	<0.001
Recipient age			0.97 (0.97–0.98)	<0.001
Transplant type (PAK/PTA *vs*. SPK)			1.30 (1.26–1.34)	<0.001
Recipient BMI			1.02 (1.01–1.03)	<0.001

^1^Adjusted for recipient age, ethnicity, BMI, HLA mismatch, PRA, transplant type, year of transplant, and the PDRI.

HR, hazard ratio; CI, confidence interval; PDRI, Pancreas Donor Risk Index; PAK, pancreas after kidney transplantation; PTA, pancreas transplantation alone; SPK, simultaneous pancreas–kidney transplantation; BMI, Body Mass Index; M → M, male donor to male recipient; F → F, female donor to female recipient; F → M, female donor to male recipient; M → F, male donor to female recipient; HLA, human leukocyte antigen; PRA, panel-reactive antibody

**Table 3:** Univariate and multivariate Cox regression analyses to evaluate the association between donor–recipient sex mismatch and pancreatic-graft failure stratified by recipient age.

	18–30 years		31–50 years		≥51 years	
	Univariate HR(95% CI) p-value	Multivariate^1^HR(95% CI) p-value	Univariate HR(95% CI) p-value	Multivariate^1^ HR(95% CI) p-value	Univariate HR(95% CI) p-value	Multivariate^1^HR(95% CI) p-value
M → M match(reference)	1.00	1.00	1.00	1.00	1.00	1.00
F → F match	1.14 (0.97–1.34) 0.108	1.07 (0.86–1.35) 0.545	1.12 (1.03–1.21) 0.011	0.96 (0.86–1.07) 0.409	0.98 (0.77–1.25) 0.868	0.78 (0.58–1.06) 0.782
F → M mismatch	1.07 (1.02–1.17) 0.041	1.12 (0.88–1.42) 0.216	1.11 (1.03–1.19) 0.009	0.99 (0.90–1.10) 0.922	1.08 (0.89–1.31) 0.439	0.95 (0.74–1.21) 0.947
M → F mismatch	1.14 (1.01–1.30) 0.043	1.27 (1.06–1.52) 0.009	1.09 (1.02–1.17) 0.011	1.07 (1.03–1.17) 0.013	1.13 (0.94–1.36) 0.206	1.07 (0.86–1.34) 0.556

^1^Adjusted for recipient BMI, ethnicity, HLA mismatch, PRA, transplant type, year of transplant and donor age, ethnicity, BMI, cause of death, donation after cardiac death, serum creatinine, hypertension history, pancreas preservation time.

HR, Hazard ratio; CI, confidence interval; M → M, male donor to male recipient; F → F, female donor to female recipient; F → M, female donor to male recipient; M → F, male donor to female recipient; BMI, Body Mass Index; HLA, human leukocyte antigen; PRA, panel-reactive antibody.
