# Supplementary material for: Influence of donor–recipient sex mismatch on long-term survival of pancreatic grafts
Source: Sci Rep. 2016 Jul 12;6:29298. doi: 10.1038/srep29298 (PMC4941418; doi:10.1038/srep29298)
Supplement: Supplementary Information [file srep29298-s1.pdf]

Title: Influence of donor–recipient sex mismatch on long-term survival of pancreatic grafts

Authors: Zhiwei Li[1], Shengmin Mei[1], Jie Xiang,[1] Jie Zhou,[1] Qijun Zhang,[1] Sheng Yan,[1,2] Lin Zhou,[1,2] Zhenhua Hu,[1,2] Shusen Zheng,[1,2*]

Supplemental Figure 1: Kaplan–Meier pancreatic-graft survival curves for sex differences between donors and recipients. Recipient sex difference (A); donor sex difference (B).

A

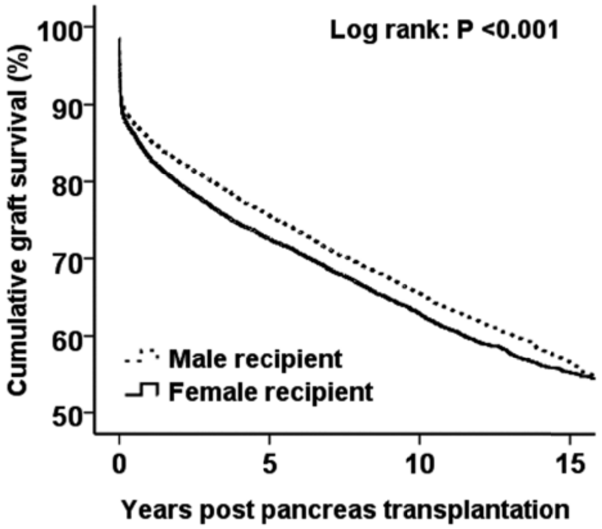

Number at risk				
Male recipients	14187	6784	2942	923
Female recipients	10008	4643	2027	635

B

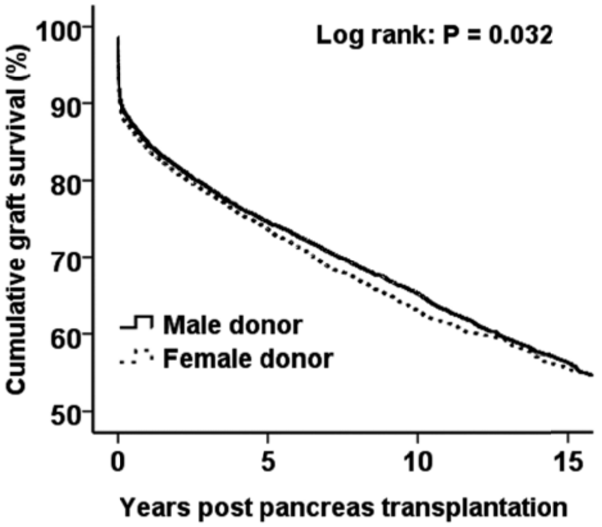

Number at risk				
Male donors	16270	7621	3302	1019
Female donors	7925	3806	1667	539

Supplemental Figure 2: Estimated pancreatic-graft survival stratified by donor–recipient sex pairing in underweight (BMI <18.5 kg/m^2), normal weight (BMI 18.5–24.9 kg/m^2), and overweight (BMI ≥25.0 kg/m^2) transplant recipients. Underweight recipients (A, D); normal-weight recipients (B, E); overweight recipients (C, F).

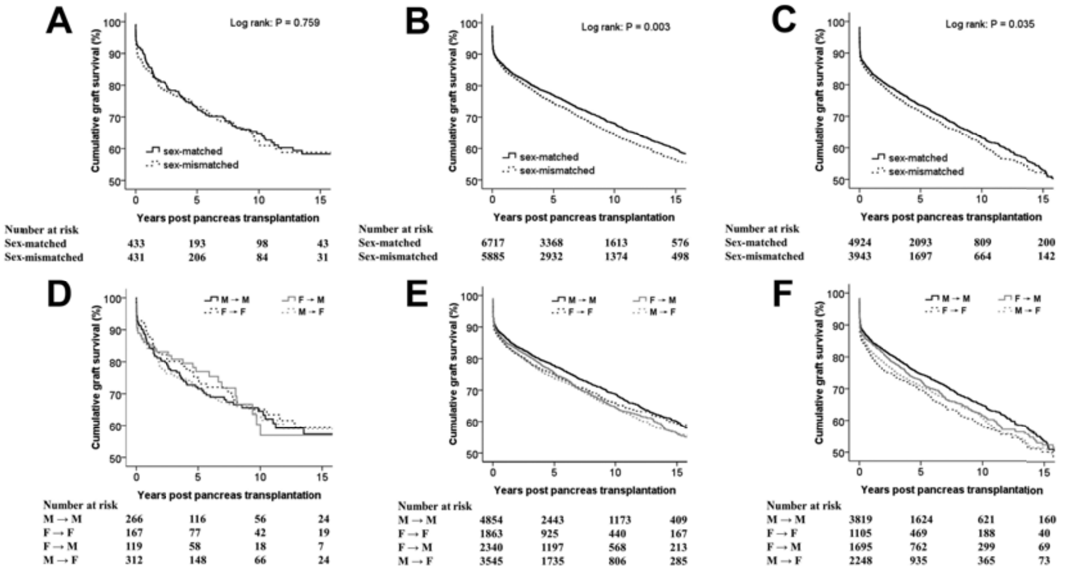

Supplemental table 1: Characteristics of pancreas donors from 1 October 1987 to 30 September 2012

	Total (n=24,195)	Male donors (n=16,270)	Female donors (n=7,925)	*P*
Age in years at organ recovery: mean (SD)	26.3 ± 10.8	24.6 ± 9.6	29.7 ± 12.3	<0.001
Ethnicity: N (%)				
Caucasian	17,691 (73.1)	11,417 (70.2)	6,274 (79.2)	<0.001
African–American	3273 (13.5)	2492 (15.3)	781 (9.9)	<0.001
Asian/Pacific Islander	393 (1.6)	229 (1.4)	164 (2.1)	<0.001
Hispanic	2700 (11.2)	2026 (12.5)	674 (8.5)	<0.001
Other	138 (0.6)	106 (0.7)	32 (0.4)	0.019
BMI: mean (SD)	25.0 ± 13.4	25.0 ± 13.2	25.0 ± 13.8	0.870
Height in centimeters: mean (SD)	171 ± 16	176 ± 15	163 ± 13	<0.001
Weight in kilograms: mean (SD)	71.3 ± 16.4	74.8 ± 16.1	64.1 ± 14.4	<0.001
Cause of death: N (%)				
Anoxia	2350 (9.7)	1342 (8.2)	1008 (12.7)	<0.001
Cerebrovascular accident/stroke	5101 (21.1)	2059 (12.7)	3042 (38.4)	<0.001
Head trauma	15,010 (62.0)	11,678 (71.8)	3332 (42.0)	<0.001
Other	1637 (6.8)	1152 (7.1)	485 (6.1)	<0.001
Donation after cardiac death: N (%)	305 (1.3)	217 (1.3)	88 (1.1)	0.159
Hypertension history: N (%)	1244 (5.1)	558 (3.4)	686 (8.7)	<0.001
Donor serum creatinine (mg/dL): mean (SD)	1.07 ± 1.05	1.14 ± 1.05	0.92 ± 1.03	<0.001
Pancreas preservation time in hours: mean (SD)	13.0 ± 5.8	13.0 ± 5.8	13.2 ± 5.7	0.012

SD, Standard deviation; BMI, Body Mass Index.

Supplemental table 2: Univariate and Multivariate Cox analyses of donor and recipient gender differences associated with pancreatic graft failure

Covariates	Univariate HR (95% CI)	*P*	Multivariate* HR (95% CI)	*P*
Male recipient (vs. female)	0.90 (0.86-0.94)	<0.001	0.96 (0.91-1.02)	0.229
Male donor (vs. female)	0.95 (0.90-0.99)	0.033	1.05 (0.98-1.12)	0.101

*Adjusted for recipient age, race, BMI, HLA mismatch, PRA, transplant type (PAK/PTA vs. SPK), year of transplant, and donor age, race, BMI, hypertension, preservation time.

HR, hazard ratio; CI, confidence interval; PDRI, pancreas donor risk index; PAK, pancreas after kidney transplantation; PTA, pancreas transplantation alone; SPK, simultaneous pancreas-kidney transplantation; BMI, body mass index; HLA, human leukocyte antigen; PRA, panel reactive antibody.

Supplemental table 3: Univariate and multivariate Cox regression analyses of donor–recipient gender mismatch associated with pancreatic graft failure, stratified by recipient BMI classes

	Underweight group		Normalweight group		Overweight group	
	Univariable HR (95% CI) p-value	Multivariable[1] HR (95% CI) p-value	Univariable HR (95% CI) p-value	Multivariable[1] HR (95% CI) p-value	Univariable HR (95% CI) p-value	Multivariable[1] HR (95% CI) p-value
M → M match (reference)	1.00	1.00	1.00	1.00	1.00	1.00
F → F match	0.94 (0.66-1.34) 0.741	0.71 (0.42-1.19) 0.190	1.10 (0.99-1.22) 0.052	0.95 (0.84-1.08) 0.437	1.24 (1.11-1.40) <0.001	0.99 (0.86-1.14) 0.846
F → M mismatch	0.96 (0.64-1.45) 0.852	0.70 (0.40-1.23) 0.216	1.11 (1.02-1.22) 0.021	1.05 (0.93-1.17) 0.456	1.11 (1.01-1.23) 0.039	0.99 (0.88-1.13) 0.983
M → F mismatch	1.03 (0.77-1.39) 0.828	1.12 (0.76-1.65) 0.577	1.15 (1.06-1.24) 0.001	1.12 (1.02-1.24) 0.023	1.17 (1.07-1.29) 0.001	1.07 (0.96-1.19) 0.240

[1]Adjusted for recipient age, ethnicity, HLA mismatch, PRA, transplant type, year of transplant and donor age, ethnicity, BMI, cause of death, donor from cardiac death, serum creatinine, hypertention history, pancreas preservation time.

BMI, body mass index; HR, hazard ratio; CI, confidence interval; M → M, male donor to male recipient; F → F, Female donor to female recipient; F → M, female donor to male recipient; M → F, male donor to female recipient; HLA, human leukocyte antigen; PRA, panel reactive antibody.

Supplemental table 4: Ten-year estimated pancreatic-graft survival for different clinical scenarios

Recipient BMI (kg/m^2)	PDRI	Transplant type	Recipient age (year)	Estimated survival probability at 10 years	95% CI
<18.5	1	SPK	18–31	0.61	(0.43, 0.79)
<18.5	1	SPK	31–50	0.70	(0.60, 0.80)
<18.5	1	SPK	≥51	0.78	(0.61, 0.95)
18.5–24.9	1	SPK	18–31	0.60	(0.55, 0.65)
18.5–24.9	1	SPK	31–50	0.71	(0.69, 0.73)
18.5–24.9	1	SPK	≥51	0.81	(0.74, 0.88)
18.5–24.9	1	PAK/PTA	18–31	0.30	(0.20, 0.40)
18.5–24.9	1	PAK/PTA	31–50	0.49	(0.43, 0.55)
18.5–24.9	1	PAK/PTA	≥51	0.57	(0.40, 0.74)
18.5–24.9	2	SPK	18–31	0.40	(0.29, 0.51)
18.5–24.9	2	SPK	31–50	0.62	(0.57, 0.67)
18.5–24.9	2	SPK	≥51	0.77	(0.68, 0.86)
18.5–24.9	2	PAK/PTA	18–31	0.20	(0.04, 0.36)
18.5–24.9	2	PAK/PTA	31–50	0.42	(0.31, 0.47)
18.5–24.9	2	PAK/PTA	≥51	0.56	(0.27, 0.85)
≥25	1	SPK	18–31	0.59	(0.50, 0.68)
≥25	1	SPK	31–50	0.69	(0.66, 0.72)
≥25	1	SPK	≥51	0.77	(0.70, 0.84)
≥25	1	PAK/PTA	18–31	0.22	(0.09, 0.35)
≥25	1	PAK/PTA	31–50	0.48	(0.42, 0.54)
≥25	1	PAK/PTA	≥51	0.66	(0.56, 0.76)
≥25	2	SPK	18–31	0.52	(0.33, 0.71)
≥25	2	SPK	31–50	0.54	(0.48, 0.60)
≥25	2	SPK	≥51	0.62	(0.50, 0.74)

BMI, body mass index; PDRI, Pancreas Donor Risk Index; CI, confidence interval; SPK, simultaneous pancreas–kidney transplantation; PAK, pancreas after kidney transplantation; PTA, pancreas transplantation alone.
